# Peripheral immune markers and amyotrophic lateral sclerosis: a Mendelian randomization study

**DOI:** 10.3389/fnins.2023.1269354

**Published:** 2023-12-21

**Authors:** Zhengwei Hu, Chunyan Zuo, Chengyuan Mao, Changhe Shi, Yuming Xu

**Affiliations:** ^1^Department of Neurology, The First Affiliated Hospital of Zhengzhou University, Zhengzhou University, Zhengzhou, China; ^2^Academy of Medical Sciences of Zhengzhou University, Zhengzhou, China; ^3^NHC Key Laboratory of Prevention and Treatment of Cerebrovascular Diseases, The First Affiliated Hospital of Zhengzhou University, Zhengzhou University, Zhengzhou, China; ^4^Henan Key Laboratory of Cerebrovascular Diseases, The First Affiliated Hospital of Zhengzhou University, Zhengzhou University, Zhengzhou, China; ^5^Institute of Neuroscience, Zhengzhou University, Zhengzhou, China

**Keywords:** amyotrophic lateral sclerosis, peripheral immunity, lymphocyte count, CD3, α-2-MRAP, C4b, IL-21, Mendelian randomization

## Abstract

**Introduction:**

The peripheral immune system changes in amyotrophic lateral sclerosis (ALS), but the causal relationship between the two is still controversial.

**Methods:**

In this study, we aimed to estimate the causal relationship between peripheral immune markers and ALS using a two-sample Mendelian randomization method. Genome-wide association study (GWAS) data on peripheral blood immune traits from European populations were used for exposure, and ALS summary statistics were used as the outcome. The causal relationship was evaluated by inverse variance weighting, MR-Egger, and weighted median methods and verified by multiple sensitivity analysis.

**Results:**

We found that the increase of one standard deviation of lymphocyte count is related to reducing ALS risk. CD3 on effector memory CD4^+^ T cell, HLA DR^+^ CD4^+^ T cell, effector memory CD8^+^ T cell, terminally differentiated CD8^+^ T cell and CD28- CD8^+^ T cell is also a protective factor for ALS. Among the circulating immune protein, the increase of one standard deviation of α-2-macroglobulin receptor-associated protein (α-2-MRAP) and C4b showed associated with low risk of ALS, while Interleukin-21 (IL-21) increases the risk of ALS.

**Discussion:**

Our study further reveals the important role of peripheral immune activity in ALS.

## 1 Introduction

Amyotrophic lateral sclerosis (ALS) is a debilitating neurodegenerative disease that is characterized by the loss of motor neurons in the spinal cord and brain, leading to muscle atrophy, and ultimately resulting in paralysis (Brown and Al-Chalabi, 2017). Unfortunately, due to the lack of effective treatment options, the disease progresses gradually, and most patients eventually succumb to respiratory failure. Despite significant research efforts, the underlying causes and mechanisms of ALS are still not fully understood (Mejzini et al., 2019).

Accumulated evidence suggests that the immune dysfunction plays a role in the progression of ALS. There is increased inflammation in the central nervous system (CNS) of patients with ALS, including reactive microglia and astrocyte activation (Liu and Wang, 2017). PET imaging with [11C]-PBR28 has also confirmed neuroinflammation and glial activation in the precentral and paracentral gyrus, the brain regions affected by ALS (Zurcher et al., 2015). It is thought that there is a crosstalk between the central immune system (CIS) and the peripheral immune system (PIS) in ALS, which has not been fully explained (Liu et al., 2020). Studies have suggested that peripheral immune cells migrate to the center, contributing to the occurrence of inflammation. Circulating immune cells, immune proteins, and cytokines are all involved in ALS (Appel et al., 2021). The total leukocyte count in the peripheral blood of ALS patients is elevated, and T cell activation is increased (Murdock et al., 2017). T cells are the main participants in adaptive immunity by recognizing antigens presented by the major histocompatibility complex (MHC) through the T cell receptor (TCR) (Murdock et al., 2017). It is generally believed that Treg cells are impaired in the peripheral immunity of ALS, and dendritic cells are also reduced in the periphery (Rusconi et al., 2017). However, CD4^+^ T and CD8^+^ T cells are all controversial, with some studies finding that CD4^+^ T cells are increased and CD8^+^ T cells are reduced in ALS, while others have found the opposite (Chen et al., 2014).

As a component of peripheral immunity, there is also a potential connection between neutrophils and ALS. An increase in neutrophil count is believed to be associated with the progression of ALS and a decrease in survival rate of ALS (Murdock et al., 2017, 2021). Compared with healthy individuals, the gene expression profile of monocytes in ALS patients has also changed, exhibiting more pronounced pro-inflammatory features and easier migration to the CNS (Zondler et al., 2016; Zhao et al., 2017). However, some studies have shown that although elevated neutrophils or monocytes are associated with lower Amyotrophic Lateral Sclerosis Functional Rating Scale – revised (ALSFRS-R) score, there is no association between it and ALS progression (Cui et al., 2022). Similarly, there is controversy over the levels of immunoglobulins and complement in the serum of ALS patients (Chen et al., 2021; Lo and Lee, 2021; Wang et al., 2021). This suggests that the peripheral immune status in ALS may be related to the disease process and thus requires further study.

To explore the relationship between peripheral immune markers and ALS, we used the Mendelian randomization (MR) approach. MR assesses the causal association between exposure and outcome and is utilized to investigate disease risk factors. Based on single nucleotide polymorphism (SNP) data from large-scale genome-wide association analysis (GWAS) of Europeans, we employed a two-sample MR analysis and found the association between immune markers and ALS.

## 2 Materials and methods

### 2.1 Exposure and instrumental variables

The immune biomarkers used for exposure include peripheral blood cell counts and ratios, circulating immune protein, blood cell surface proteins, etc., referred to a previous report (Lindbohm et al., 2022). The complete list is available in Supplementary Table S1. In order to apply the MR approach, instrumental variables (IVs) were selected based on the following three assumptions: (i) the variables being used as instruments are associated with the exposure of interest; (ii) the variables should not be related to any confounders factors between the exposure and the outcome; (iii) the variables are only associated with the outcome through its effect on the exposure. To meet the relevance assumption, SNPs that met the significance threshold *p* < 5.00e-8 in the exposure GWAS data were selected as IVs. IVs were clumped according to the 1,000 Genomes Project linkage disequilibrium (LD) structure, and independent instruments (*R*^2^ < 0.001 with any other associated SNP within 250 kb) with the lowest *p* value were retained to ensure the independence assumption of MR. If no SNPs in the GWAS summary data of ALS satisfied the above criteria, proxy SNPs strongly correlated with exposure (*R*^2^ > 0.8) were selected.

### 2.2 Outcome

The outcome summary data was sourced from a large-scale GWAS study of Europeans, including 27,205 ALS patients and 110,881 controls, totaling 138,086 individuals. All the patients were diagnosed with ALS according to the revised El Escorial Criteria (van Rheenen et al., 2021). The exposure and outcome data were harmonized to align the effect alleles.

### 2.3 Two-sample Mendelian randomization analysis

The two-sample MR analysis was conducted using the TwoSampleMR package (version 0.5.6) in R software (version 4.2.1). When IV satisfied the core assumptions, inverse variance weighting (IVW) was used as the main method to investigate the causal association between exposure and outcome, and the MR-Egger and weighted median (WM) methods were also applied. Cochran’s Q statistics were conducted to test IVs heterogeneity, with no heterogeneity determined when *p* > 0.05. MR-Egger intercept and MR-PRESSO global test were used to examine the existence of pleiotropy. MR-PRESSO global test was employed using the MR-PRESSO package (version 1.0) (Verbanck et al., 2018). When horizontal pleiotropy exists, the MR-PRESSO outlier test was used to correct it. Leave-one-out (LOO) analysis was used to evaluate if the results were driven by a single SNP. Since the outcome is a binary variable, odd ratio (OR) was used to represent causality. Phenotypic variance R^2^ was calculated based on a previous study (Papadimitriou et al., 2020). To assess the presence of weak IV bias, F-statistic was calculated using the formula *F* = *R*^2^ (*N* − 2)/(1 − *R*^2^). SNPs with *F* < 10 are considered to be weak IVs. Statistical power analysis for exposure was performed using the publicly available tool mRnd.^1^ The type I error rate was set at 0.05. The results of multiple comparisons were corrected using FDR correction.

## 3 Results

### 3.1 Causal relationship of immune markers and ALS risk

**Figure 1:**
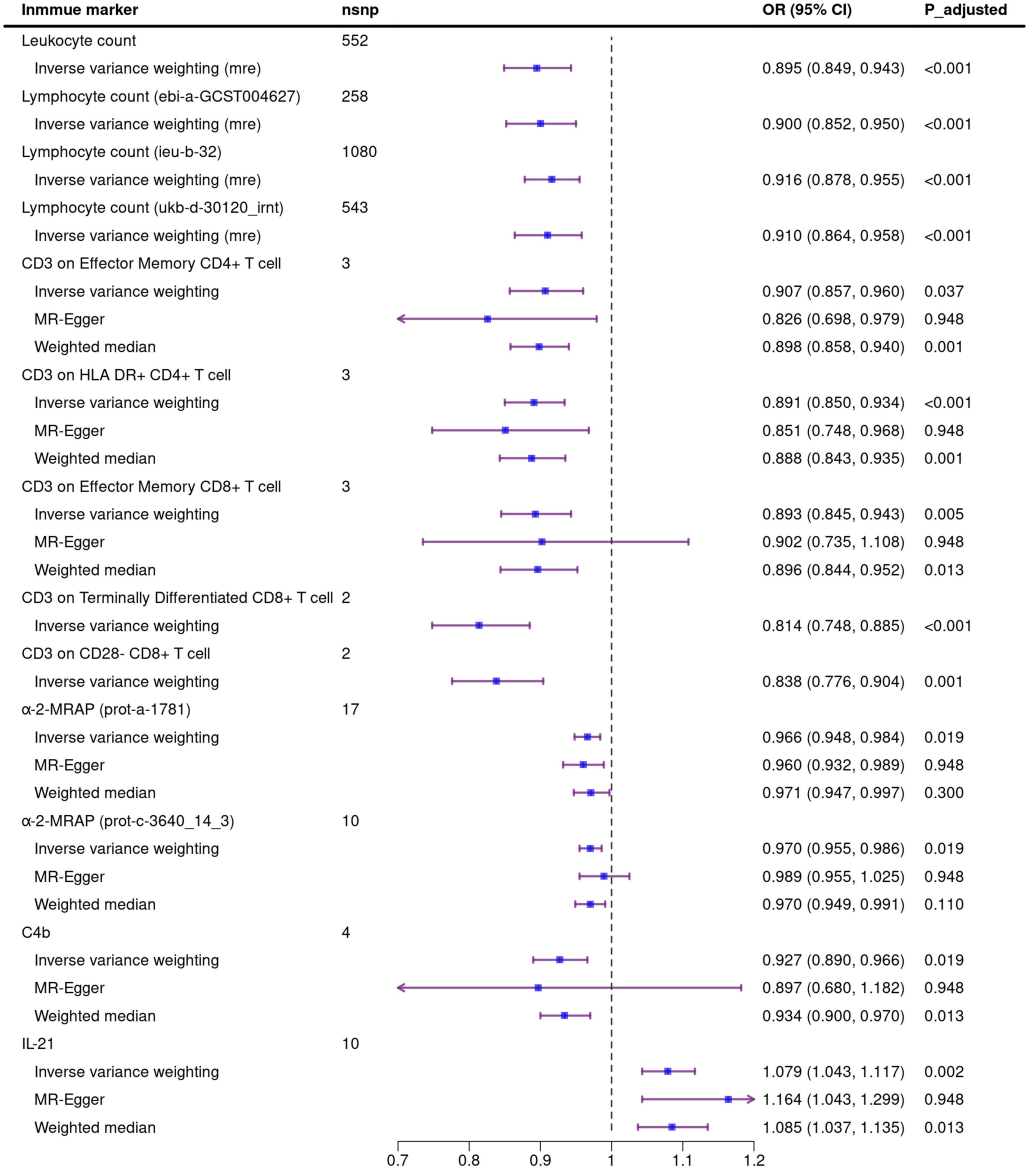
Mendelian randomization analysis of the causal association between immune markers and amyotrophic lateral sclerosis. Due to the existence of heterogeneity in the four exposures about leukocyte count and lymphocyte count, the random effect model was used in IVW method. Nsnp, number of single nucleotide polymorphism; OR, odds ratio; CI, confidence interval; mre, random effect model.

A total of 1,140 immune markers were analyzed for their association with ALS using three MR methods. After FDR correction, 12 immune traits associated with the reduced risk of ALS were observed using the IVW method, including four about peripheral immune cell count: leukocyte count [OR: 0.895, 95%CI: (0.849, 0.943), *P*_adjusted = 0.005], lymphocyte count [ebi-a-GCST004627, OR:0.900, 95%CI: (0.852, 0.950), *P*_adjusted = 0.012; ieu-b-32, OR: 0.916, 95%CI: (0.878, 0.955), *P*_adjusted = 0.005; ukb-d-30120_irnt, OR: 0.910, 95%CI: (0.864, 0.958), *P*_adjusted = 0.019]; five immunocyte surface markers: CD3 on effector memory CD4^+^ T cell [OR: 0.907, 95%CI: (0.857, 0.960), *P*_adjusted = 0.037], CD3 on HLA DR^+^ CD4^+^ T cell [OR: 0.891, 95%CI: (0.850, 0.934), *P*_adjusted <0.001], CD3 on effector memory CD8^+^ T cell [OR: 0.893, 95%CI: (0.845, 0.943), *P*_adjusted = 0.005], CD3 on terminally differentiated CD8^+^ T cell [OR: 0.814, 95%CI: (0.748, 0.885), *P*_adjusted < 0.001], CD3 on CD28^−^ CD8^+^ T cell [OR: 0.838, 95%CI: (0.776, 0.904), *P*_adjusted = 0.001]; three immune related proteins in circulation: α-2-macroglobulin receptor-associated protein [α-2-MRAP, prot-a-1781, OR: 0.966, 95%CI: (0.948, 0.984), *P*_adjusted = 0.019; prot-c-3640_14_3, OR: 0.970, 95%CI: (0.955, 0.986), *P*_adjusted = 0.019], C4b [OR: 0.927, 95%CI: (0.890, 0.966), *P*_adjusted = 0.019]. In addition, we also identified that Interleukin-21 (IL-21) is associated with an increased risk of ALS [OR: 1.079, 95%CI: (1.043, 1.117), *P*_adjusted = 0.002]. The causal estimates of immune markers on ALS risk are summarized in Figure 1.

### 3.2 Sensitivity analysis

We performed sensitivity analysis on MR results. In the Cochran’s Q test, there is heterogeneity between SNPs in the four exposures about leukocyte or lymphocyte count. We used the random effect model to estimate the amount of MR effect and found a causal relationship between these exposure and ALS (Figure 1). The heterogeneity analysis results were consistent for the other exposures, and no significant difference was found. In the analysis of exposure leukocyte count and lymphocyte count (ebi-a-GCST004627), horizontal pleiotropy was found in MR Egger regression intercept, and no significant outliers in MR-PRESSO distortion test. For the other two exposures of lymphocyte counts, although MR Egger regression showed horizontal pleiotropy, the MR-PRESSO analysis distortion test showed robust results (Table 1). We did not detect SNPs that disproportionately affect the effect estimates in LOO analysis. Due to the insufficient number of SNPs, the LOO analysis of “CD3 on Terminally Differentiated CD8^+^ T cell” and “CD3 on CD28^−^ CD8^+^ T cell” cannot be performed (Supplementary Figures S1–S12).

**Table 1:** Sensitivity analysis for immune markers that showed significant correlations with ALS.

Immune marker	Cochran’s Q		MR Eggr		MR.PRESSO		
	*Q*	*p*	Intercept	*p*	Global test *p*	Distortion test *p*	*p* (outlier-corrected)
Leukocyte count	736	<0.001	0.003	0.024	<0.001^†^	na^⁑^	na^⁑^
Lymphocyte count (ebi-a-GCST004627)	345.139	<0.001	0.005	0.023	<0.001^†^	na^⁑^	na^⁑^
Lymphocyte count (ieu-b-32)	1407.652	<0.001	<−0.001	0.849	<0.001^†^	0.949	<0.001
Lymphocyte count (ukb-d-30120_irnt)	685.914	<0.001	0.003	0.079	<0.001^†^	0.904	<0.001
CD3 on effector memory CD4^+^ T cell	3.752	0.153	0.034	0.461	na^‡^	–	–
CD3 on HLA DR^+^ CD4^+^ T cell	0.574	0.75	0.017	0.588	na^‡^	–	–
CD3 on effector memory CD8^+^ T cell	1.044	0.593	−0.004	0.933	na^‡^	–	–
CD3 on terminally differentiated CD8^+^ T cell	0.895	0.344	na^‡^	na^‡^	na^‡^	–	–
CD3 on CD28^−^ CD8^+^ T cell	0.132	0.716	na^‡^	na^‡^	na^‡^	–	–
α-2-MRAP (prot-a-1781)	16.436	0.423	−0.006	0.614	0.486	–	–
α-2-MRAP (prot-c-3640_14_3)	6.323	0.707	−0.024	0.27	0.731	–	–
C4b	5.974	0.113	0.014	0.831	0.236	–	–
Interleukin-21	6.068	0.733	−0.027	0.192	0.736	–	–

^†^The MR PRESSO outlier test was performed due to the existence of horizontal pleiotropy.

^‡^The test was not conducted due to not enough instrumental variables.

^⁑^No significant outliers in MR.PRESSO Distortion test.

## 4 Discussion

In this study, we used the two-sample Mendelian randomization method to examine the causal relationship between 1,140 immune markers and ALS risk. We identified twelve immune traits negatively correlated with ALS risk, including leukocyte count, lymphocyte count, CD3 on effector memory CD4^+^ T cell, CD3 on HLA DR^+^ CD4^+^ T cell, CD3 on effector memory CD8^+^ T cell, CD3 on terminally differentiated CD8^+^ T cell, CD3 on CD28^−^ CD8^+^ T cell, α-2-MRAP and C4b. Besides, we found that a one-standard deviation increase of IL-21 was associated with a higher risk of ALS.

Dysfunction of the immune system may be one of the causes of clinical heterogeneity in ALS. At different stages of ALS, the same immune cells may play positive or negative roles. The total number of white blood cells in the peripheral blood of ALS patients increases and tends to be higher over time (Murdock et al., 2017; Cui et al., 2022). A previous Mendelian randomization study showed a negative causal relationship between white blood cell count and ALS (Li et al., 2020). In our study, although we found a negative causal relationship between leukocyte count and ALS using random effects model of IVW method, the bias introduced by pleiotropy cannot be removed. This may be due to we used the newly published GWAS data with larger population size. The central premise for using Mendelian randomization for causal inference is the absence of horizontal pleiotropy. We used the MR-PRESSO outlier test to eliminate abnormal SNPs (outliers) and estimate the corrected results. However, there were no significant outliers in the white blood cell count and one lymphocyte count (ebi-a-GCST004627). Nevertheless, we still found that two exposures about lymphocyte count were not horizontally pleiotropic after correction. This result proves that the absolute value of lymphocytes has a protective effect on ALS.

CD3 is a general marker present on T cell surfaces and functions as a crucial component of the TCR complex, which plays an essential role in the development and activation of T cells (Letourneur and Klausner, 1992). TCR recognizes endogenous or exogenous antigens, and CD3 facilitates T cell activation and proliferation by building a bridge between the recognized antigens and effector signals. T cells are core participants in adaptive immunity, specifically recognizing antigens presented by MHC and TCR complexes (Shah et al., 2021). In ALS, activated effector T cells cross the blood–brain barrier and infiltrate the brain and spinal cord (Engelhardt et al., 1993). CD4^+^ T cells are generally considered a protective factor for ALS, recognizing antigens presented by MHC II through antigen-presenting cells (APC). Like memory CD8^+^ T cells, memory CD4^+^ T cells can quickly expand and differentiate into effector cells upon encountering the same antigen, providing a faster and more effective immune response than naïve T cells. HLA-DR labeled CD4^+^ T cells with effector characteristics (Tippalagama et al., 2021). CD8^+^ T cells receive antigens presented by MHC I molecules and exert cytotoxic effects. In the late stage of ALS, CD8^+^ T cells have been found to infiltrate the central nervous system. Memory CD8^+^ T cell is divided into three subgroups. Central memory CD8^+^ T cell exhibit longer steady-state proliferation and pluripotency, as well as the ability to recall secondary lymphoid organs. Effect memory CD8^+^ T cells possess greater cytotoxicity, but the recall ability is limited. Terminally differentiated CD8^+^ T cell has high expression of lysosomal enzyme genes and granzymes and provides the most substantial protective effect on cells (Milner et al., 2020). Our results show that CD3 on two types of memory CD8^+^ T cells has a negative causal relationship with ALS. The loss of CD28 is associated with chronic antigen stimulation. CD28^−^CD8^+^ T cells are a class of regulatory cells that regulate T cells directly and indirectly. This type of cell has been seen to inhibit the severity of the disease in multiple sclerosis models (Chen et al., 2018; Ceeraz et al., 2021). However, the role of these CD4^+^ or CD8^+^ T cell subsets in ALS has not been fully understood. We speculate the activation state indicated by CD3 may be related to the protective effect of these cells in ALS.

The complement system regulates innate immune responses and also combines with adaptive immunity. Evidence suggests that the complement pathway is involved in ALS. For example, C3 and C4 are elevated in the serum of ALS patients (Kjaeldgaard et al., 2018). In addition, elevated levels of C1q and C4 were also observed in the spinal cord and motor cortex of ALS patients (Sta et al., 2011). We found that C4 may be a protective factor for ALS, suggesting that further research is needed on the role of complement in ALS. We also found that alpha-2-macroglobulin receptor-associated protein (α-2-MRAP, RAP) may have a protective effect on ALS. α-2-MRAP, also known as LRPAP1, is an endoplasmic reticulum-resident protein highly expressed in the kidneys and multiple brain regions. α-2-MRAP has a high affinity with LRP, preventing the binding of all other ligands and affecting the level of low-density lipoprotein cholesterol (LDL-C) (Zhang et al., 2017). An MR analysis showed elevated lipid levels are associated with an increased risk of ALS (Xia et al., 2023). However, although research shows that α-2-MRAP is associated with susceptibility to dementia, its relationship with ALS has not been studied.

IL-21 can be produced by follicular helper T cells (T_FH_), peripheral helper T cells (T_PH_), and Th17 cells. IL-21 acts on B cells to produce high-affinity, class-switched antibodies and also acts on CD8^+^ T cells, affecting differentiation and metabolism. Under the reflection of CD8^+^ T cells to chronic antigen presentation, IL-21 may be involved in driving autoimmune diseases (Ren et al., 2021). IL-21 is elevated in the serum of AD and MCI patients, and blocking IL-21 can alleviate neuroinflammation and Aβ plaques in 5 × FAD mice (Agrawal et al., 2022). Although a meta-analysis showed that peripheral blood IL-1β, IL-6, and IL-8 were elevated in ALS patients (Hu et al., 2017), there is limited understanding of the relationship between IL-21 and ALS. Since the immune dysregulation present in ALS, the regulatory effects of IL-21 on immune cells may play a role in reducing ALS risk.

Previous observational studies have focused on the relationship between immune factors and diagnosed ALS. In this study, we explored the causal relationship between multiple peripheral immune markers and ALS. However, limitations also exist. The immunological characteristics measured may be influenced by the temporary conditions at the time of detection and may not reflect the genetically determined immune status. The exposure and outcome data we used were from European populations, and the results may not be applicable to other racial groups. Nonetheless, based on the Mendelian randomization results of large-scale GWAS summary data, we demonstrated the causal relationship between peripheral immune markers and ALS risk. As a systemic, immune-mediated disease, intensive research on these peripheral immune traits will help to discover the complex mechanism of immune response in ALS.

## Data availability statement

The original contributions presented in the study are included in the article/Supplementary material, further inquiries can be directed to the corresponding authors.

## Author contributions

ZH: Data curation, Formal analysis, Investigation, Visualization, Writing – original draft. CZ: Data curation, Formal analysis, Investigation, Visualization, Writing – original draft. CM: Data curation, Validation, Writing – review & editing. CS: Writing – review & editing, Conceptualization, Funding acquisition, Methodology, Project administration. YX: Conceptualization, Funding acquisition, Methodology, Project administration, Writing – review & editing.

## Footnotes

^1^https://shiny.cnsgenomics.com/mRnd/
