# Supplementary material for: Peripheral immune markers and amyotrophic lateral sclerosis: a Mendelian randomization study
Source: Front Neurosci. 2023 Dec 21;17:1269354. doi: 10.3389/fnins.2023.1269354 (PMC10768049; doi:10.3389/fnins.2023.1269354)
Supplement: Supplementary file 1 [file Table_1.DOCX]

**Supplementary figure 1.** Leave-one-out (LOO) plot for single SNP effect size of causal effects for leukocyte count (ukb-d-30000_irnt) on ALS.

**Supplementary figure 2.** Leave-one-out (LOO) plot for single SNP effect size of causal effects for lymphocyte count (ebi-a-GCST004627) on ALS.

**Supplementary figure 3.** Leave-one-out (LOO) plot for single SNP effect size of causal effects for lymphocyte count (ieu-b-32) on ALS.

**Supplementary figure 4.** Leave-one-out (LOO) plot for single SNP effect size of causal effects for lymphocyte count (ukb-d-30120_irnt) on ALS.

**Supplementary figure 5.** Leave-one-out (LOO) plot for single SNP effect size of causal effects of immune markers on ALS. (A), CD3 on Effector Memory CD4+ T cell. (B), CD3 on HLA DR+ CD4+ T cell. (C), CD3 on Effector Memory CD8+ T cell. (D), alpha-2-macroglobulin receptor-associated protein (prot-a-1781). (E), alpha-2-macroglobulin receptor-associated protein (prot-c-3640_14_3). (F), C4b. (G), IL-21.

**Supplementary figure 6.** Funnel plot for the relationship between the SNP effect size of causal immune traits and the corresponding effect size estimates of ALS. (A), leukocyte count (ukb-d-30000_irnt). (B), lymphocyte count (ebi-a-GCST004627). (C), lymphocyte count (ieu-b-32). (D), lymphocyte count (ukb-d-30120_irnt). (E), CD3 on Effector Memory CD4+ T cell. (F), CD3 on HLA DR+ CD4+ T cell. (G), CD3 on Effector Memory CD8+ T cell. (H), CD3 on Terminally Differentiated CD8+ T cell. (I), CD3 on CD28- CD8+ T cell. (J), alpha-2-macroglobulin receptor-associated protein (prot-a-1781). (K), alpha-2-macroglobulin receptor-associated protein (prot-c-3640_14_3). (L), C4b. (M), IL-21.

**Supplementary figure 7.** Forest plot of causal effects for leukocyte count (ukb-d-30000_irnt) on ALS.

**Supplementary figure 8.** Forest plot of causal effects for lymphocyte count (ebi-a-GCST004627) on ALS.

**Supplementary figure 9.** Forest plot of causal effects for lymphocyte count (ieu-b-32) on ALS.

**Supplementary figure 10.** Forest plot of causal effects for lymphocyte count (ukb-d-30120_irnt) on ALS.

**Supplementary figure 11.** Forest plot of causal effects for immune markers on ALS. (A), CD3 on Effector Memory CD4+ T cell. (B), CD3 on HLA DR+ CD4+ T cell. (C), CD3 on Effector Memory CD8+ T cell. (D), CD3 on Terminally Differentiated CD8+ T cell. (E), CD3 on CD28- CD8+ T cell. (F), alpha-2-macroglobulin receptor-associated protein (prot-a-1781). (G), alpha-2-macroglobulin receptor-associated protein (prot-c-3640_14_3). (H), C4b. (I), IL-21.

**Supplementary figure 12.** Scatter plot for the relationship between the SNP effect size of causal immune markers and the corresponding effect size estimates of ALS. (A), leukocyte count (ukb-d-30000_irnt). (B), lymphocyte count (ebi-a-GCST004627). (C), lymphocyte count (ieu-b-32). (D), lymphocyte count (ukb-d-30120_irnt). (E), CD3 on Effector Memory CD4+ T cell. (F), CD3 on HLA DR+ CD4+ T cell. (G), CD3 on Effector Memory CD8+ T cell. (H), CD3 on Terminally Differentiated CD8+ T cell. (I), CD3 on CD28- CD8+ T cell. (J), alpha-2-macroglobulin receptor-associated protein (prot-a-1781). (K), alpha-2-macroglobulin receptor-associated protein (prot-c-3640_14_3). (L), C4b. (M), IL-21.
