# Supplementary figures and images for: Peripheral immune markers and amyotrophic lateral sclerosis: a Mendelian randomization study
Source: Front Neurosci. 2023 Dec 21;17:1269354. doi: 10.3389/fnins.2023.1269354 (PMC10768049; doi:10.3389/fnins.2023.1269354)

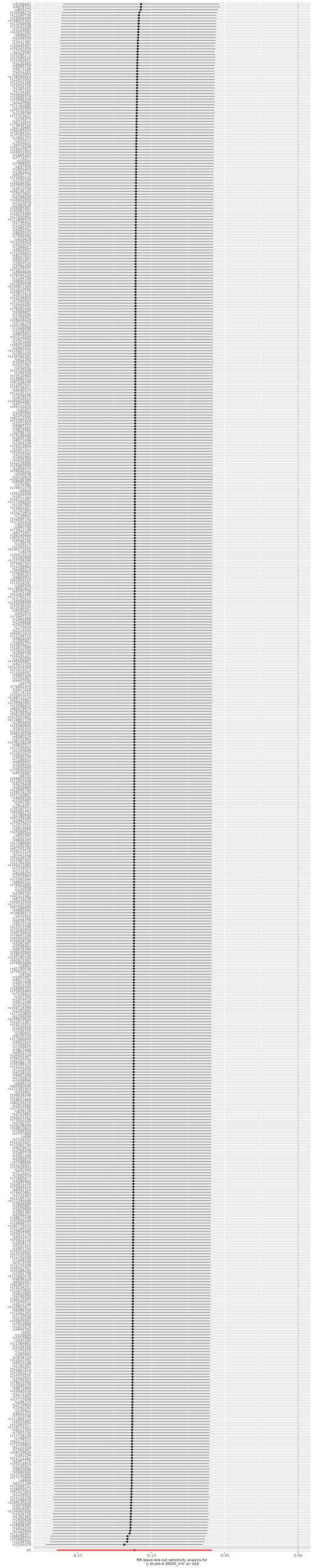

Supplement: Supplementary file 5 [file Image_1.TIF]

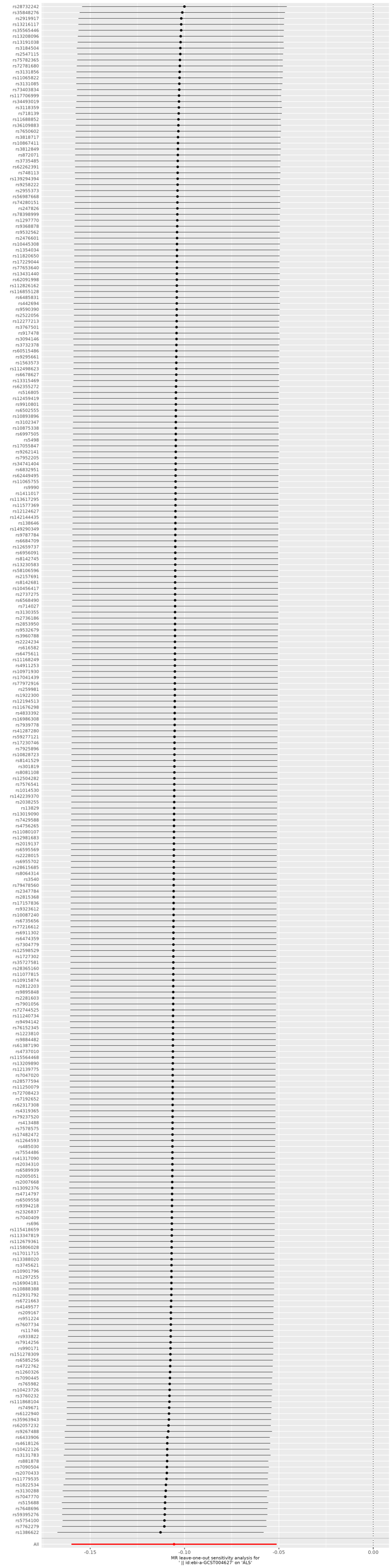

Supplement: Supplementary file 6 [file Image_2.TIF]

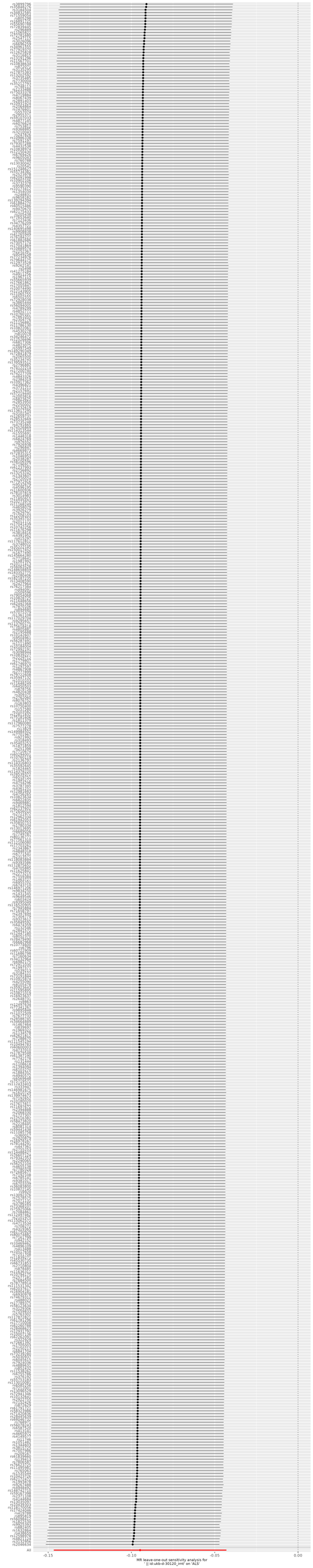

Supplement: Supplementary file 8 [file Image_4.TIF]

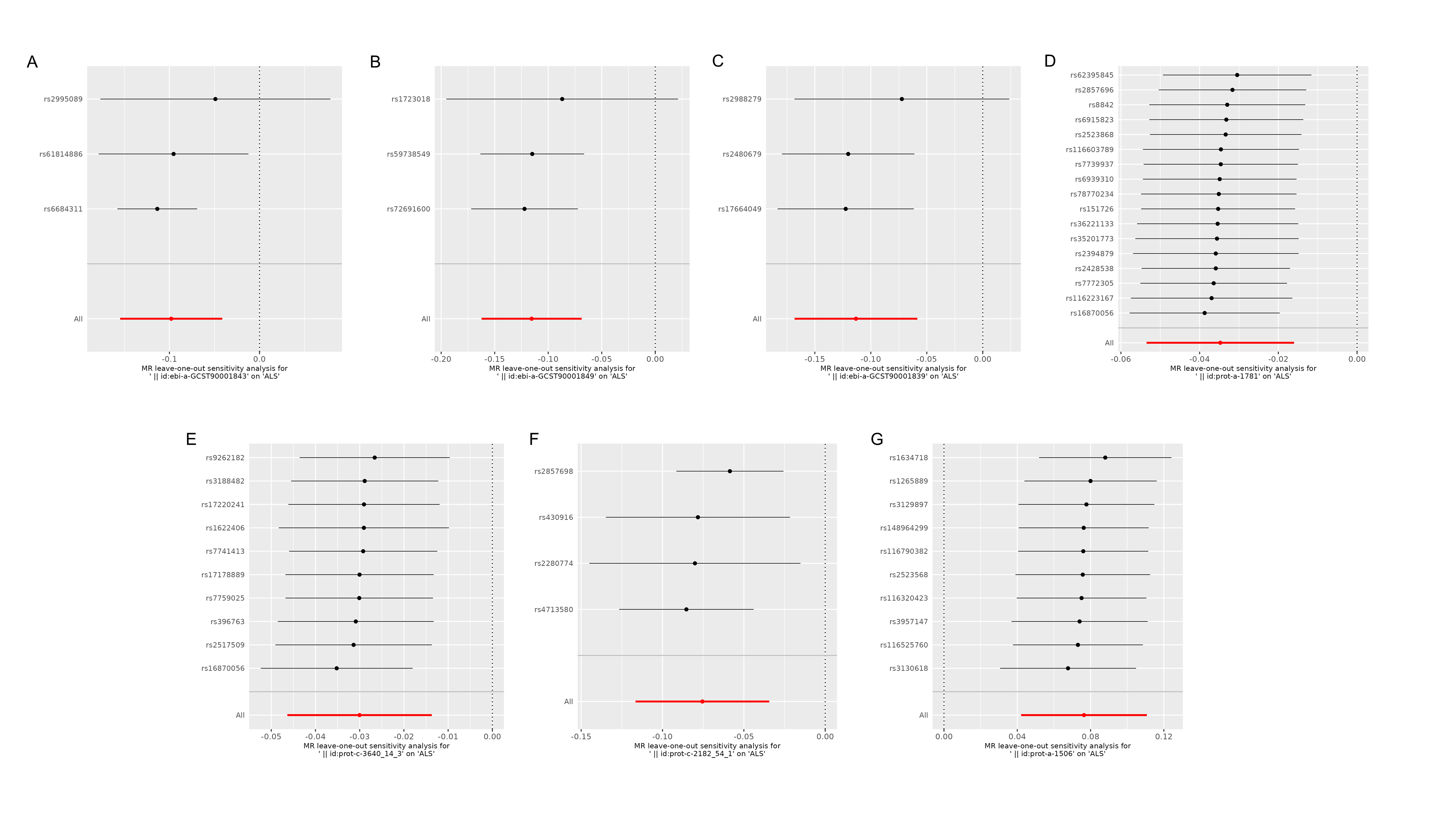

Supplement: Supplementary file 9 [file Image_5.TIF]

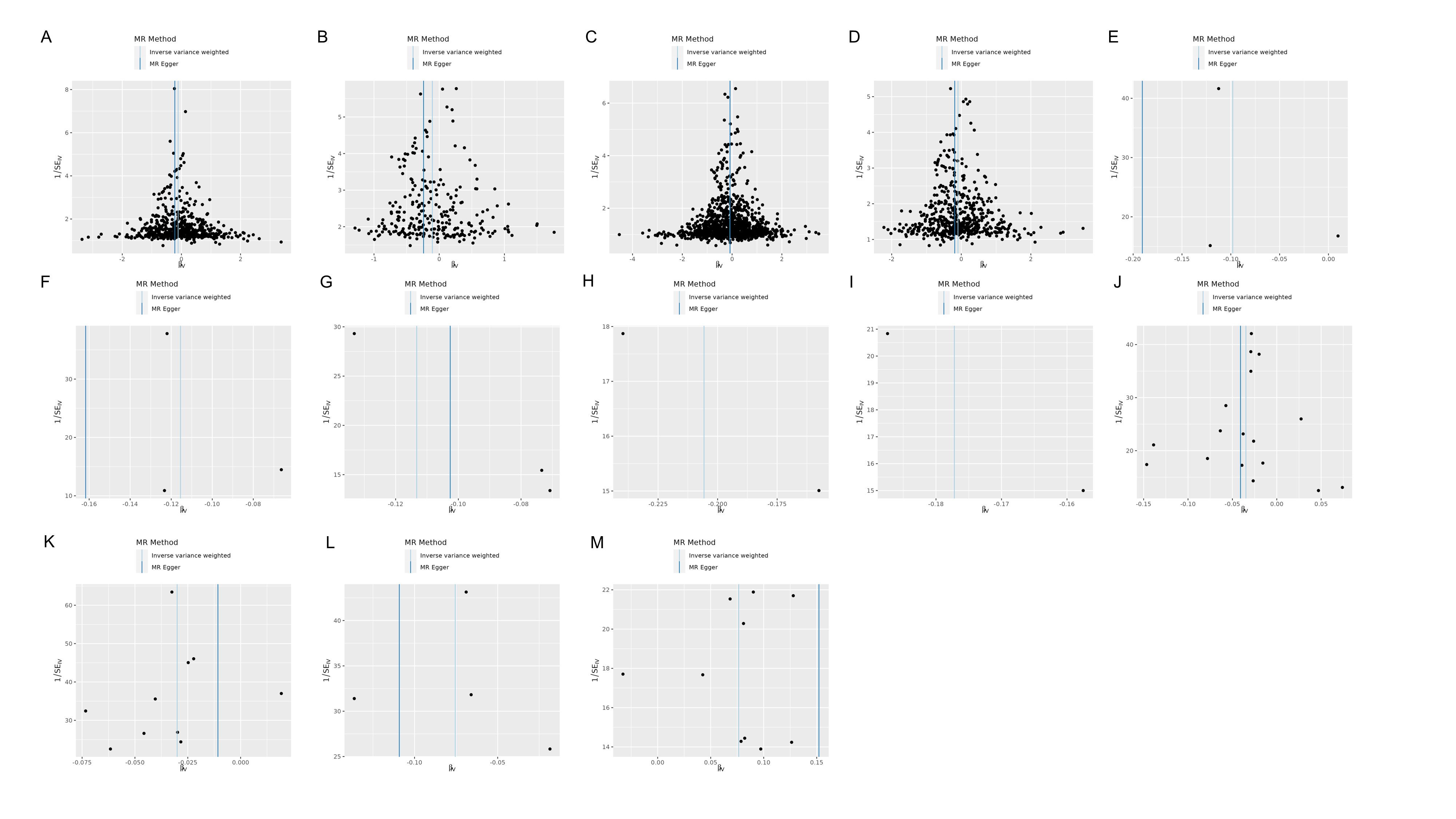

Supplement: Supplementary file 10 [file Image_6.TIF]

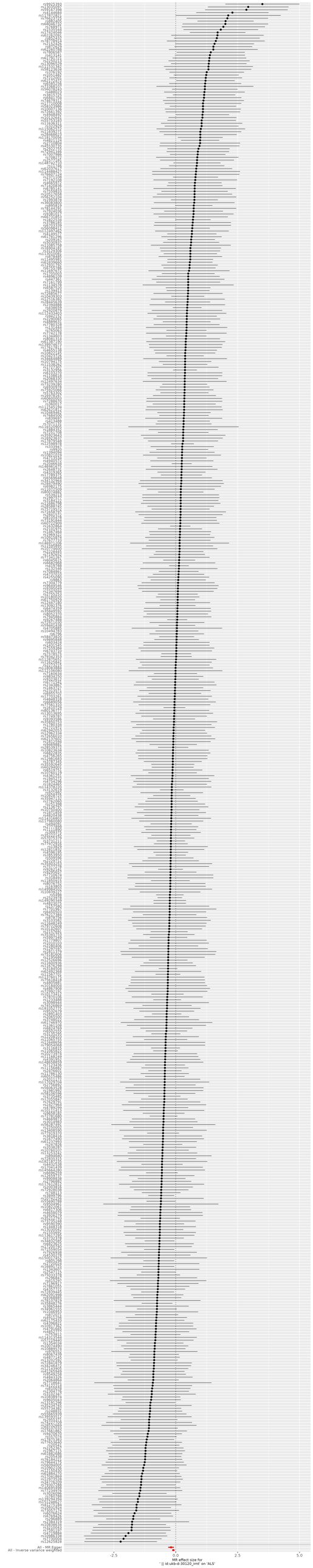

Supplement: Supplementary file 11 [file Image_7.TIF]

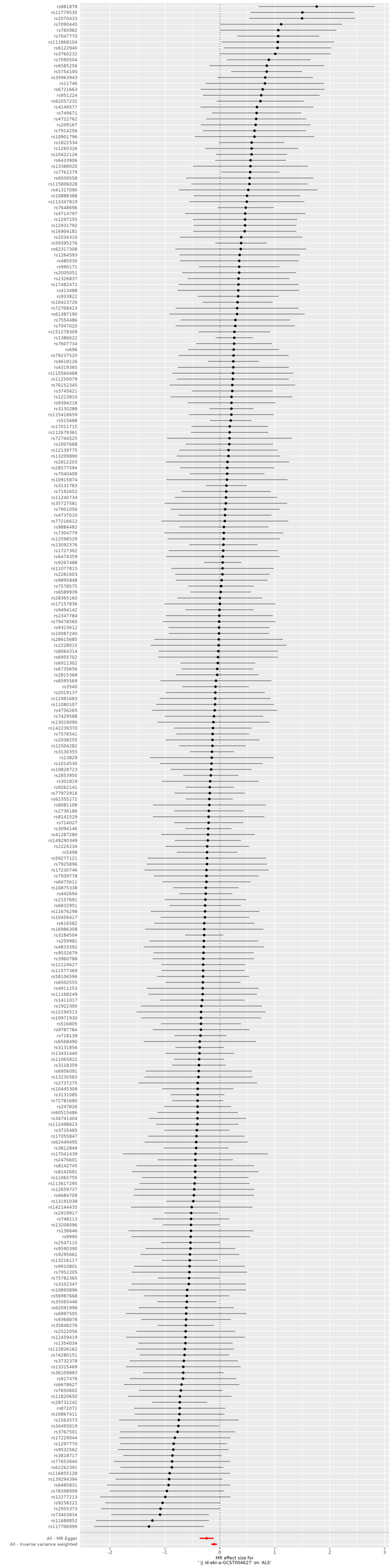

Supplement: Supplementary file 12 [file Image_8.TIF]

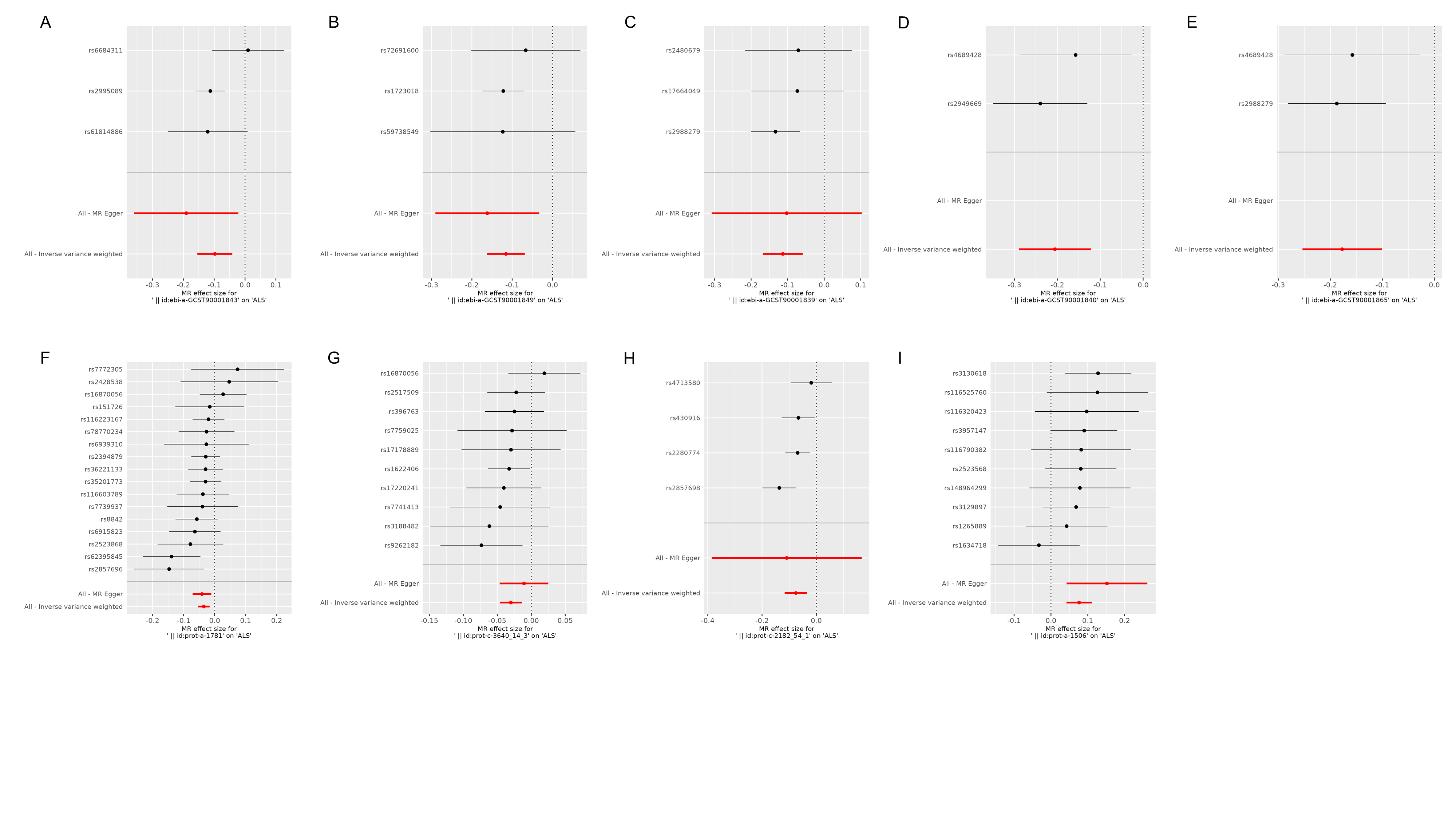

Supplement: Supplementary file 15 [file Image_11.TIF]

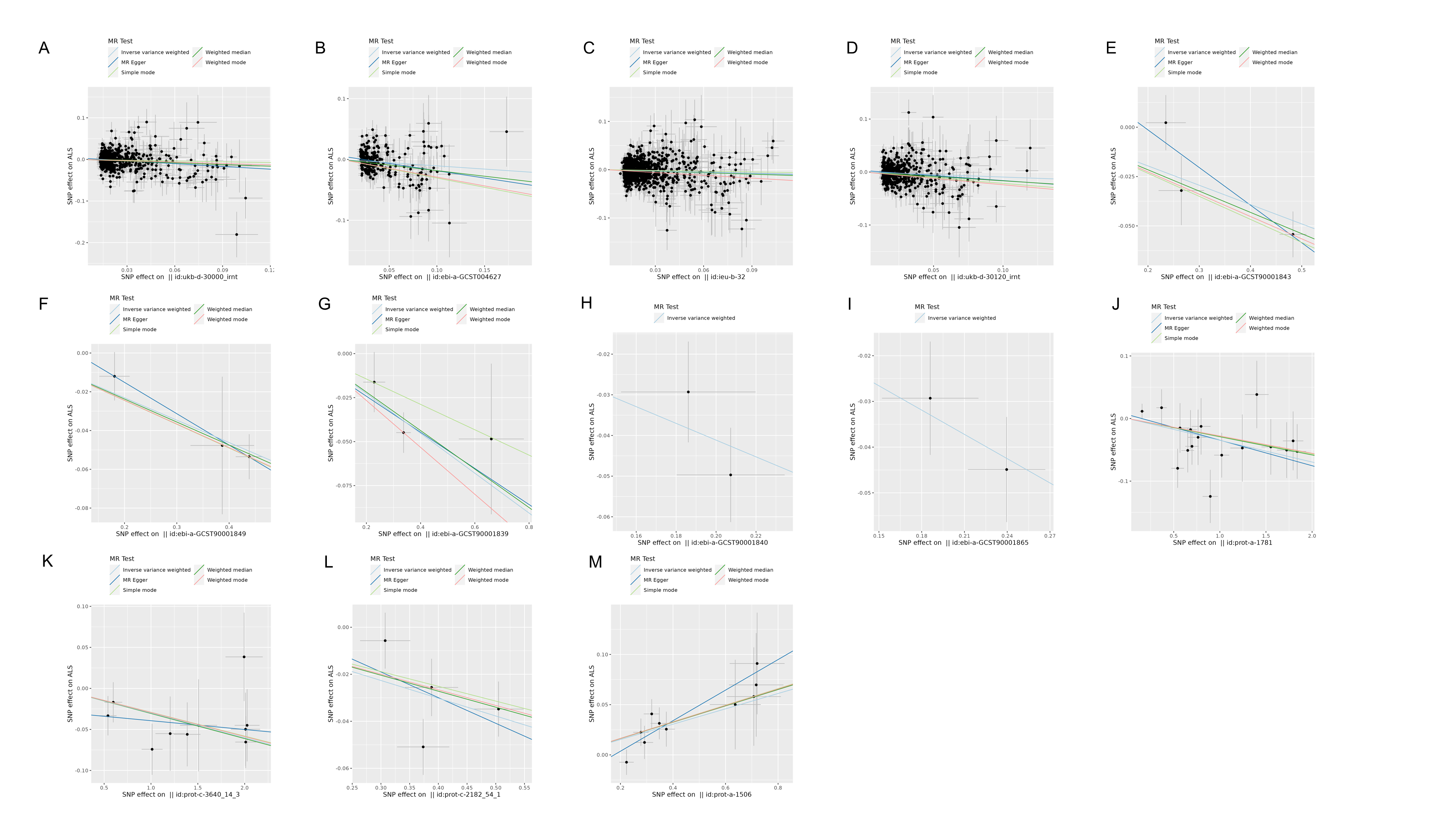

Supplement: Supplementary file 16 [file Image_12.TIF]
